# Plasma miR-199a-5p is increased in neutrophilic phenotype asthma patients and negatively correlated with pulmonary function

**DOI:** 10.1371/journal.pone.0193502

**Published:** 2018-03-05

**Authors:** Yali Huang, Shengding Zhang, Xiaoyu Fang, Lu Qin, Yu Fan, Dandan Ding, Xiansheng Liu, Min Xie

**Affiliations:** 1 Department of Respiratory and Critical Care Medicine, Tongji Hospital, Tongji Medical College, Huazhong University of Science and Technology, Wuhan, Hubei, People's Republic of China; 2 Department of Respiratory Medicine, The Third People's Hospital of Wuhan, Wuhan, Hubei, People's Republic of China; National Yang-Ming University, TAIWAN

## Abstract

**Objective:**

We investigated the relationship between plasma miRNAs levels and inflammatory characteristics in asthmatic patients.

**Methods:**

Eligible adults with untreated asthma (n = 35) underwent a clinical assessment, sputum induction, and assessment of pulmonary function test and Asthma Control Test (ACT) scores. Asthma phenotypes were defined using the sputum cell count. miR-199a-5p expression was measured using quantitative real-time polymerase chain reaction (qPCR). Lipopolysaccharide (LPS) stimulation was used to detect miR-199a-5p secretion from peripheral blood-derived neutrophil, lymphocyte, macrophage and BEAS-2B cells. The correlation of miR-199a-5p expression with clinical parameters was analyzed using multiple linear regression analysis. In silico analysis predicted the target genes and signaling pathway of miR-199a-5p. Transfection of miR-199a-5p mimics in human airway smooth muscle cells (HASMCs) was performed in vitro.

**Results:**

The miRNA-199a-5p levels in plasma and sputum increased significantly in patients with neutrophilic asthma compared to healthy subjects (*ps* = 0.014 and 0.006, respectively). Expression of miR-199a-5p in the plasma of asthmatic patients positively correlated with sputum miR-199a-5p expression (r = 0.511, *p* = 0.021). The miR-199a-5p level was only elevated with LPS stimulation in neutrophils but not macrophages, lymphocytes, or epithelial cells from healthy controls (*p* < 0.01). miR-199a-5p expression increased in response to LPS (*p* = 0.005) and LPS combined with IL-4 (*p* = 0.003), but not IL-4 alone. However, peripheral neutrophils from eosinophilic asthma patients did not respond to LPS with increased miR-199a-5p expression (n = 5, *p* > 0.05) in contrast to the significant response from neutrophilic patients (n = 4, *p* < 0.0001). miR-199a-5p negatively correlated with FEV_1,_ FVC and PEF (r = -0.377, *p* = 0.026; r = -0.419, *p* = 0.012; and r = -0.392, *p* = 0.024, respectively). Multivariate correlation analysis confirmed that the plasma miR-199a-5p levels negatively correlated with FEV_1_ in patients with asthma (Adjusted R^2^ = 0.164, *p* = 0.015). In silico analysis suggested that the WNT signaling pathway participates in miR-199a-5p mediation of smooth muscle cell hypertrophy. In vitro experiment, miR-199a-5p mimics inhibited the protein expressions of WNT2 and WNT4, decreased the c-myc expression and dramatically increased the Sm-MHC expression in HASMCs.

**Conclusion:**

Plasma miR-199a-5p was increased in neutrophilic asthma and negatively correlated with pulmonary function, which suggests that miR-199a-5p actively contributes to disease pathogenesis by modulating the inflammatory process and transferring the signal from inflammatory cells to structure cells.

## Introduction

Asthma is featured with chronic airway inflammation and encompasses various phenotypes with distinct clinical characteristics, triggers and inflammatory processes [1, 2]. It is difficult to characterize the diverse biological processes that determine these observed phenotypes using current diagnostic tools, and asthma is still not completely understood. There is an urgent need to identify noninvasive biomarkers to characterize specific phenotypes and predict their responses to treatment.

MicroRNAs (miRNAs) are a class of endogenous noncoding single-stranded short (20–25 nucleotides) RNAs. MicroRNAs function as sequence-specific negative regulators by interacting with the 3'-untranslated region of target mRNAs, which leads to mRNA cleavage or translational repression [3, 4]. miRNAs also indirectly affect gene expression via holistic effects on methylation and regulation of transcription factor expression [5]. Therefore, miRNAs play a critical role in regulating various biological processes and these factors are involved in the pathogenesis of a variety of diseases, including asthma [6, 7]. Previous studies suggested important roles for the let-7 family, miR-21, miR-146, and miR-155 in regulating pathophysiology by controlling T-cell function and cytokine production, which play roles in the inflammatory response of asthma [8–12].

miRNAs are produced by a broad spectrum of cells and secreted into body fluids, such as plasma / serum, saliva, bronchoalveolar lavage fluid, cerebrospinal fluid and urine [13–16]. The miRNAs in plasma and other body fluids are stable in enclosed vesicles termed exosomes or in combination with HDL and Ago2. miRNAs exert biological effects through body fluid circulation and may serve as noninvasive biomarkers for the diagnosis and prognosis in a variety of diseases, including asthma [17–20].

Most studies focus on the role of intracellular specific miRNAs in asthmatic patients, but several studies investigated circulation miRNAs as biomarkers for asthma in recent years. Panganiban et al. revealed an up-regulation of miRNA-1248, which targets IL-5, in the serum of 10 asthmatic patients compared to control subjects [21]. Changes in miRNA expression were reported in the plasma of children with asthma, including up-regulation of miR-3162-3p, which was significantly associated with MEF25, and up-regulation of miR-1260a, which correlated with the treatment schedule of these patients [22]. Another miRNA profiling study of sera of asthmatic children also demonstrated a T_H_2 cytokine–promoting role of miR-21 in patients with steroid-resistant asthma [23]. Panganiban et al. identified 30 miRNAs (detectable in the plasma) that were differentially expressed between healthy, allergic, and asthmatic subjects in an independent study. They also suggested that circulating miRNAs can be used to differentiate between asthma phenotypes [24]. Milger K et al. recently suggested that distinct plasma miRNAs were differentially expressed in human allergic asthma and associated with patient clinical characteristics [25]. Studies on the serum of children with asthma revealed that reduced circulatory miRNA expression was associated with an increase in PC20 [26]. However, to our knowledge, no study has linked miRNA expression in the plasma of patients with asthma to neutrophil phenotypes, of which a large proportion lack a corticosteroid response and require extra medical care.

This study performed miRNA gene expression analysis on induced sputum and plasma from patients with asthma and associated miRNA expression with inflammatory phenotypes. We demonstrated that miR-199a-5p expression is increased in patients with neutrophilic asthma and associated with pulmonary function reduction, which suggests that plasma miR-199a-5p contributes to inflammation and mechanical processes in patients with neutrophilic asthma.

## Materials and methods

### Subjects

We recruited all voluntary patients during June 15, 2015-April 20, 2016. Adults with asthma (n = 35) and healthy subjects (n = 15) were enrolled from the Department of Respiratory and Critical Care Medicine at Tongji Hospital (Wuhan, China) and Huazhong University of Science and Technology, China. The following inclusion criteria were used for asthma patients: asthma was diagnosed according to Global Initiative for Asthma (GINA) [27] with methacholine PC20 < 2.5 mg or bronchodilation FEV1 change > 200 ml and 12%; had not received any corticosteroid treatment in the last 3 months; and successfully induced sputum, of which 18 cases were eosinophilic (sputum eosinophils ≥ 3% and sputum neutrophils < 61%) and 17 cases were neutrophilic (sputum neutrophils ≥ 61% and sputum eosinophils < 3%). The following exclusion criteria were used for asthmatics: acute episode; respiratory infection in the last 2 weeks; pregnancy; bronchiectasis; other respiratory disease; and serious organ failure. Healthy controls had no history of chronic respiratory disease and were not atopic. All participants were nonsmokers (never smokers or ex-smokers who quit smoking for longer than 6 months and had a smoking history of less than 5 pack years). Atopy was defined as at least 1 specific IgE (≥ 0.35 kUI/L) toward common aeroallergens, a positive skin prick test response, or both. The Ethics Review Board of Tongji Hospital approved the study, which conformed to the declaration of Helsinki. All participants provided written informed consent prior to clinical data and sample collection. Subject samples were identified using study numbers rather than names. Personally identifiable information will not be revealed to anyone who is not a research team member. Additional details are available in the S1 File.

### Sputum induction

Sputum was induced using hypertonic saline (4.5%) and processed using DTT (Sigma-Aldrich Corporation, St. Louis, MO, USA) as previously described [28]. Briefly, whole sputum was weighed, and 2 volumes of 0.1% DDT were added. The sample was homogenized via rotation for 30 minutes at room temperature and filtered through 40-mm nylon gauze. Samples were centrifuged (500 g) for 10 minutes at 4°C to separate the cell and supernatant. Cells were resuspended at 1×10^6^/mL, and cytospins were prepared using a cytocentrifuge. Cells were stained (Wright-Giemsa), and a differential cell count was obtained from 400 non-squamous cells. Asthmatic patients were categorized according to the differential cell count of induced sputum [29]. The remaining cells were stored in RNA later (Life Technologies, Carlsbad, CA, USA) at -80°C until extraction for gene expression analysis.

### Blood sample collection

Whole blood (5 mL) was collected in EDTA tubes from each subject, and plasma was separated via centrifugation at 1600 g for 10 minutes at 4°C followed by centrifugation at 16,000 g for 10 min at 4°C to remove all cell debris. The plasma supernatant was collected and stored at -80°C until further analysis.

### RNA extraction

Total RNA was extracted from 400 μl of plasma or each sputum cell pellet using the mirVana™ PARIS™ Kit (Ambion, Foster City, CA, USA) according to the manufacturer’s instructions. Each RNA sample was quantified using a spectrophotometer (NanoDrop 1000, Thermo Scientific, Wilmington, DE, USA).

### Validation using quantitative real-time PCR (qRT-PCR)

Reverse transcription was performed using the PrimeScript RT reagent Kit (Takara, Dalian, China). The specific miRNA reverse and PCR primers were synthesized by Riobio Co. Ltd. (Guangzhou, China). Real-time PCR assays were performed using the SYBR Premix ExTaq (Takara, Dalian, China) on a 7500 Real-Time PCR System (Life Technologies, Carlsbad, CA, USA). Data were analyzed using automatic settings to assign the baseline. The Ct value was defined as the fractional cycle number at which the fluorescence passed a given threshold. The miRNA expression levels were normalized to U6. Sm-MHC mRNA expression level of HASMCs was normalized to beta-actin. The relative expression levels were calculated using the 2^-Δct^ method.

### miR-199a-5p expression following LPS stimulation in peripheral blood and BEAS-2B cells

Blood was obtained from healthy controls and patients with neutrophilic or eosinophilic asthma. Peripheral blood cell subsets were sorted using the Ficoll-Paque and sticking wall method as described previously [30–32], and mononuclear, neutrophil and lymphocytic cells were isolated. Briefly, whole blood was mixed with 6% hydroxyethyl starch (TBD sciences, Tianjin, China) to allow for erythrocyte sedimentation. The upper layer with blood cells was collected and added to the liquid surface of the Human peripheral blood lymphocyte separation solution (TBD sciences, Tianjin, China) followed with centrifuging (600 g) for 30 minutes. Then, cells were collected for neutrophils in the lower layer and mononuclear in the layer between the plasma and the separation fluid. Neutrophils were purified by red blood cell lysis buffer (TBD sciences, Tianjin, China), identified with Wright-Giemsa stain and immediately followed with treatment as described below. The peripheral blood mononuclear cells were seeded at 2×10^6^ cells/well in 12-well plates in RPMI 1640 culture medium (Hyclone, UT, USA) supplemented with 10% fetal bovine serum (FBS) (Gibco, Australia) and 40 ng/ml recombinant human GM-CSF (peprotech, Princeton, USA) for two hours. After the cells sticking wall, lymphocytes in the medium suspension was collected and identified with Wright-Giemsa stain. Mononuclear cells adhered to the wall were fed every other day with RPMI 1640 culture medium supplemented with 10% FBS and 40 ng/ml recombinant human GM-CSF and split 1:1 every 4 days. After 10 days, human macrophages were characterized according to morphology and immunohistochemical stain with CD68. Trypan blue staining was used to count the alive cells. The purity and survival rate of all the three cell types were greater than 90% (S1 Fig).

Neutrophils and lymphocytes were resuspended in RPMI 1640 culture medium (Hyclone, CT, USA) with 10% FBS and seeded at 0.5×10^6^ cells per well of 24 well plates immediately after isolation. While macrophages were washed extensively with PBS, digested with trypsin, and seeded at 0.25×10^6^ cells per well of 24 well plates for further treatment. Peripheral blood cell subsets and BEAS-2B cells were treated with LPS (100 ng/mL) (Sigma-Aldrich Corporation, St. Louis, MO, USA) and/or IL-4 (10 ng/mL) (peprotech, Princeton, USA) for 24 h. The cell-free supernatant and cell pellets were collected and stored for further analysis. We tried to measure miR-199a-5p levels both in the cell homogenates and in the supernatants of cell cultures, but the level of miR-199a-5p in supernatant was too low to be detected. MiR-199a-5p levels in cell homogenates were used for further comparison analysis.

### miR-199a-5p mimics transfection in HASMCs

The primary HASMCs were obtained from ScienCell Research Laboratories, San Diego, CA, USA, and grown in DMEM (HyClone, CT, USA) supplemented with 10% fetal bovine serum (Gibco, Australia) according to the manufacturer’s guidelines. Cells were used at the fourth to seventh passage. For miR-199a-5p overexpression, 50nM of miR-199a-5p mimics or negative miRNA control (Riobio, Guangzhou, China) were transfected into cells using Lipofectamine 3000 (Life Technologies, Carlsbad, CA, USA). After 48 hours of transfection, cells were collected for further protein (WNT2, WNT4 and c-myc) and mRNA (Sm-MHC) analysis.

### Western blot analysis

Total cellular protein was extracted using RIPA lysis buffer (Aspenbio, Wuhan, China) and were measured by the BCA protein assay kit (Aspenbio, Wuhan, China). The primary antibodies including WNT2 (1:500 dilution, Proteintech, Wuhan, China), WNT4 (1:1000 dilution, Abgent, San Diego, CA, USA), and c-myc (1:500 dilution, Proteintech, Wuhan, China) were incubated in 4°C overnight. Goat anti-rabbit IgG (1:4000 dilution, SAB, College Park, MD, USA) and Goat anti-mouse IgG (1:4000 dilution, Aspenbio, Wuhan, China) were used as secondary antibodies. The protein expression levels were normalized to β-actin (1:4000, Sungenebiotech, Tianjin, China).

### Statistical analysis

Measurement Data are presented as the means ± SDs and medians and interquartile ranges when appropriate, enumeration data are expressed as the number and/or rate. Variables in groups were compared using nonparametric tests for skewed distributions (Kruskal-Wallis test). Parametric tests (ANOVA test) were performed for normal distributions and similar variances between groups. The correlation coefficients were calculated using test of Spearman's rank-correlation coefficient and Multiple linear regression analysis. P values less than 0.05 were considered statistically significant. The data were analyzed using SPSS statistics software (Version 17, IBM, Chicago, IL, USA).

## Results

### Demographic characteristics

Table 1 shows the demographic, functional, and sputum characteristics of the 50 participants, including 15 healthy control subjects and 35 asthmatic patients (18 patients with eosinophilic asthma and 17 patients with neutrophilic asthma). There were no significant differences in age, sex, BMI, smoking status, or pack years between the 3 groups. Patients with asthma were more likely to be atopic, with higher IgE levels than the healthy control group. Patients with eosinophilic asthma exhibited significantly increased blood eosinophil counts compared to the other 2 groups and higher FeNO levels compared to patients with neutrophilic asthma. Lower FEV_1_ and FEV_1_/FVC ratios were observed in patients with eosinophilic asthma compared to healthy subjects. Patients with neutrophilic asthma exhibited a significantly increased percentage of neutrophils and reduced proportion of macrophages in sputum compared to the other 2 groups. The percentage of sputum eosinophils was significantly higher in asthma patients with the eosinophilic phenotype.

**Table 1:** Clinical characteristics of participants.

	Healthy controls	Patients with eosinophilic asthma‡	Patients with neutrophilic asthma‡	*p* value#
No.	15	18	17	
Demographic characteristics				
Age (y)	35(27–44)	40(26–48)	42(28–52)	0.476
Sex M/F, no./no.(%/%)	5/10(33/67)	6/12(33/67)	4/13(24/76)	0.774
BMI (kg/m^2^)	22.2±2.7	23.0±2.9	21.5±2.1	0.280
Atopy,* no. (%)	0(0)	6(33)§	6(35)§	0.019
Ex-smoker,※ no. (%)	0(0)	3(17)	2(12)	0.352
Functional characteristics				
Blood eosinophils(×10^9^)	0.10±0.04	0.41±0.25‖	0.21±0.20 †	<0.001
Serum IgE(IU/mL)	34.1(20.4–41.2)	147.9(31.1–355.2) §	125.6(82.5–237.2) §	0.009
FEV_1_ (L)	3.2(2.7–3.3)	2.6(2.2–3.3)	2.6(2.0–3.2)	0.199
FEV_1_ (%)	101.8(88.8–110.8)	86.6(77.9–90.9) §	94(80.7–105.2)	0.014
FVC (L)	3.8(3.5–4.0)	3.5(3.0–4.7)	3.4(2.4.5)	0.600
FVC(%)	105.5(93.4–111.7)	106.2(94.5–113.0)	103.8(98.7–117.7)	0.842
FEV_1_/FVC ratio (%)	81.7(76.8–86.7)	71.0(66.9–74.5)‖	75.6(65.0–83.4)	0.002
FeNO (ppb)	ND	51.9(23.8–105.7)	24(11–44.5)	0.036
ACT score	ND	16(15–18)	17(16–19)	0.290
Sputum characteristics				
Macrophages (%)	40.2(31.5–44.5)	36.8(21.0–55.2)	6.9(3.7–18.2)‖**¶**	<0.001
Neutrophils (%)	51.3(50.0–54.6)	25.2(15.1–35.3) ‖	86.4(70.4–90.5)‖**¶**	<0.001
Eosinophils (%)	0.2(0–0.8)	12.5(7.4–34.9)‖	0.97(0–2.05)**¶**	<0.001

Data are presented as distributions (yes/no), means ± SDs, or medians with IQRs. Analysis was performed using Pearson x2 tests or the Kruskal-Wallis test, followed by the Mann-Whitney U test.

M, male; F, female; BMI, body mass index; FENO, fraction of exhaled nitric oxide; ACT, Asthma Control Test; IQR, interquartile range; ND, not determined.

*Atopy was defined as at least 1 specific IgE toward common aeroallergens, a positive skin prick test response, or both.

※Ex-smoker was defined as not smoking for at least six months.

‡Sputum eosinophilic inflammation, ≥ 3% eosinophils and < 61% neutrophils; neutrophilic inflammation, ≥ 61% neutrophils and < 3% eosinophils.

# *p* value, overall *p* value between three groups.

§, *p* < 0.05 versus healthy subjects.

‖, *p* < 0.001 versus healthy subjects.

†, *p* < 0.01 versus patients with eosinophilic asthma.

¶, *p* < 0.001 versus patients with eosinophilic asthma.

### Plasma miRNA-199a-5p expression profiles in different phenotypes of asthmatic patients

**Fig 1:**
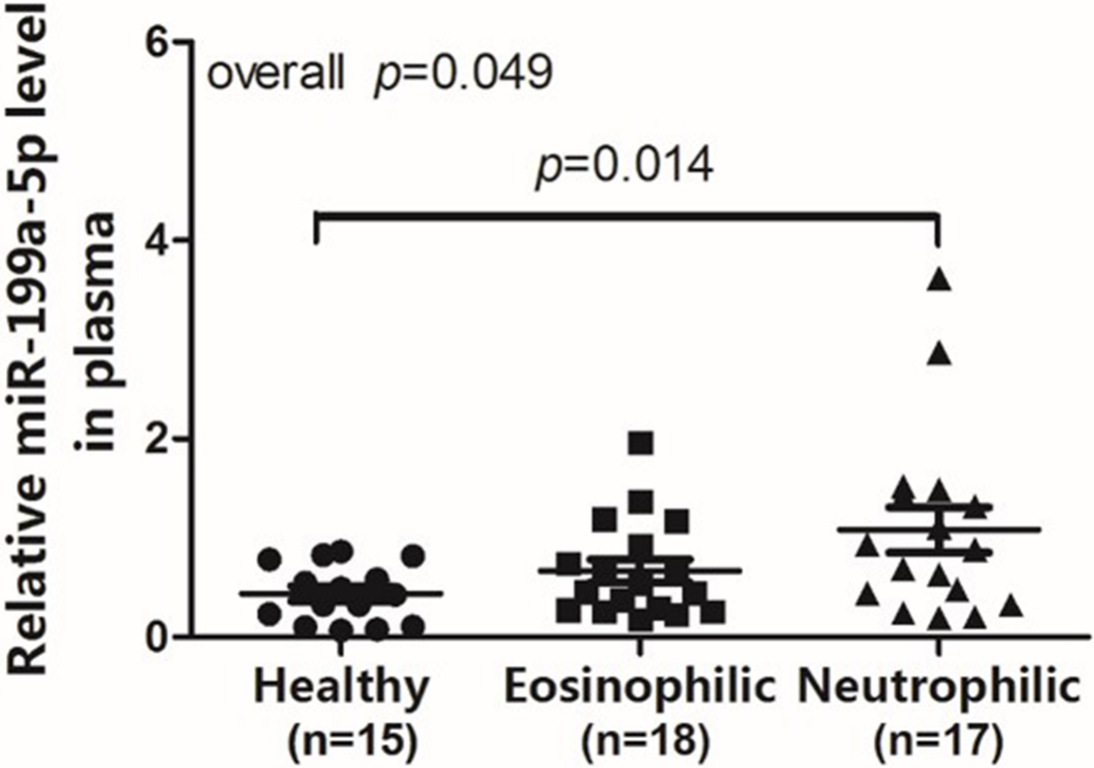
Relative miR-199a-5p levels in the plasma of different groups. Normalized miR-199a-5p expression levels in the plasma of different inflammatory phenotypes and healthy controls. miR-199a-5p expression levels were normalized to U6.

The plasma miRNA-199a-5p levels differed between groups (overall *p* = 0.049) and increased significantly in patients with neutrophilic asthma compared to healthy subjects (*p* = 0.014). There was no significant difference between eosinophilic asthmatics and healthy subjects (Fig 1).

### Plasma miRNA-199a-5p correlated with the sputum miRNA-199a-5p expression profile

**Fig 2:**
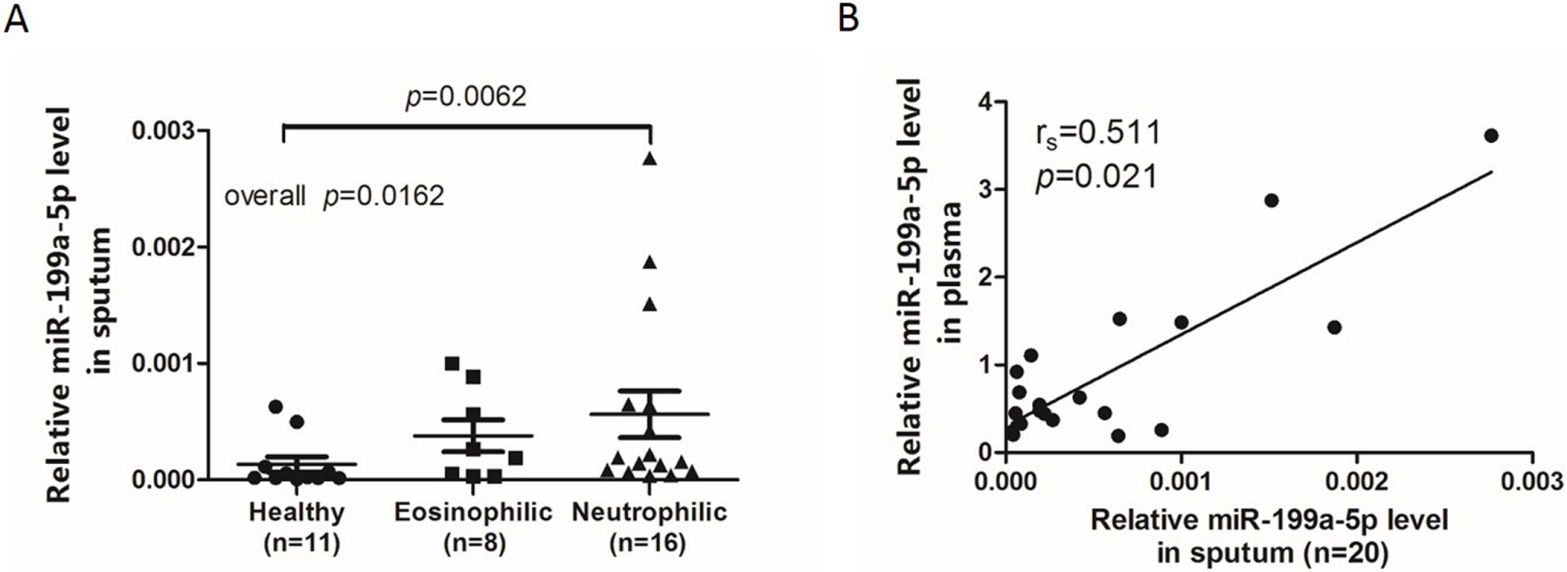
Relative miR-199a-5p levels in sputum in different groups. (A) Normalized miR-199a-5p expression levels in sputum from different inflammatory phenotypes and healthy controls. (B) The correlation between the miR-199a-5p expression levels in plasma and sputum. Data are presented as dot plots with fitted regression lines. The miR-199a-5p expression levels were normalized to U6. Spearman R-value and p-value are indicated.

Previous studies demonstrated increased let-7 levels in plasma and decreased levels in the epithelium of asthmatic patients, which indicates that plasma miRNA does not necessarily parallel local expression. We detected sputum miRNA-199a-5p using qRT-PCR. The results demonstrated that the quantitative expression of miRNA-199a-5p in sputum was significantly higher in patients with neutrophilic asthma compared to healthy controls (Fig 2A). miR-199a-5p expression in the plasma of asthmatic patients positively correlated with expression in sputum (Fig 2B Spearman ' s rank correlation coefficient, r = 0.511, *p* = 0.021).

### Neutrophils secreted miR-199a-5p following LPS stimulation, but not macrophages, lymphocytes or epithelium cells

**Fig 3:**
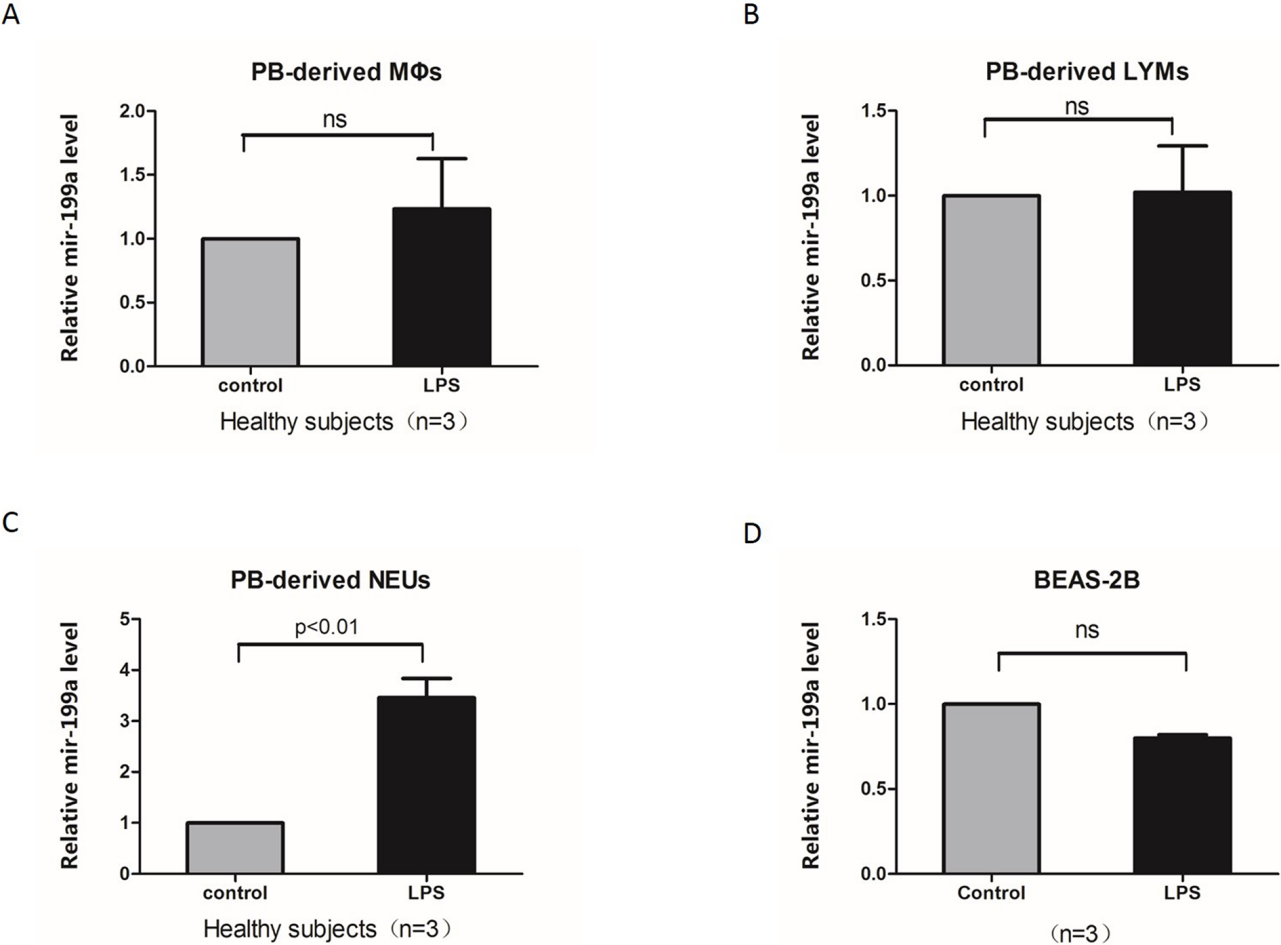
miR-199a-5p expression levels from different cells following LPS stimulation. (A-C) miR-199a-5p expression levels in PB-derived macrophages (A), lymphocytes (B) and neutrophils (C) isolated from three healthy subjects treated or untreated with LPS (100 ng/mL) for 24 hours. (D) miR-199a-5p expression levels in BEAS-2B cell lines, untreated or treated with LPS (100 ng/mL) for 24 hours. Relative miR-199a-5p levels were calculated over untreated controls.

We examined whether the miR-199a-5p levels increased in human peripheral blood cell subsets and an epithelial cell line (BEAS-2B). Peripheral blood cells were separated and treated with LPS for 24 h. Changes in miR-199a-5p expression in response to LPS for each individual’s peripheral blood cell subsets and BEAS-2B cells were compared to its own untreated control. The miR-199a-5p levels were only elevated in neutrophils from healthy controls (*p* < 0.01). The fold change was not significant following LPS stimulation in peripheral blood cell-derived macrophages, lymphocytes, or epithelial cells (Fig 3).

### Neutrophils from neutrophilic asthma patients secreted miR-199a-5p following LPS stimulation but not from eosinophilic asthma patients

**Fig 4:**
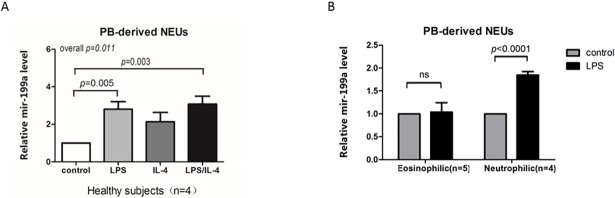
miR-199a-5p expression levels in PB-derived neutrophils isolated from healthy subjects and asthmatic patients. (A) miR-199a-5p expression levels in PB-derived neutrophils isolated from four healthy subjects treated with LPS (100 ng/mL) and/or IL-4 (10 ng/mL) for 24 hours. (B) miR-199a-5p expression levels in PB-derived neutrophils isolated from six asthmatic patients of different inflammatory phenotypes, untreated or treated with LPS (100 ng/mL), for 24 hours. Relative miR-199a-5p levels were calculated over untreated controls.

Human peripheral blood neutrophils from healthy controls were treated with LPS with/without IL-4 for 24 h. The results demonstrated increased miR-199a-5p expression in response to LPS (*p* = 0.005) and LPS combined with IL-4 (*p* = 0.003), but not IL-4 alone (Fig 4A). However, peripheral neutrophils from eosinophilic asthma patients did not respond to LPS by secreting miR-199a-5p (n = 5, *p* > 0.05), which contrasts the significant response in neutrophilic patients (n = 4, *p* < 0.001, Fig 4B).

### Correlation of the plasma miR-199a-5p levels with clinical characteristics and pulmonary function

**Fig 5:**
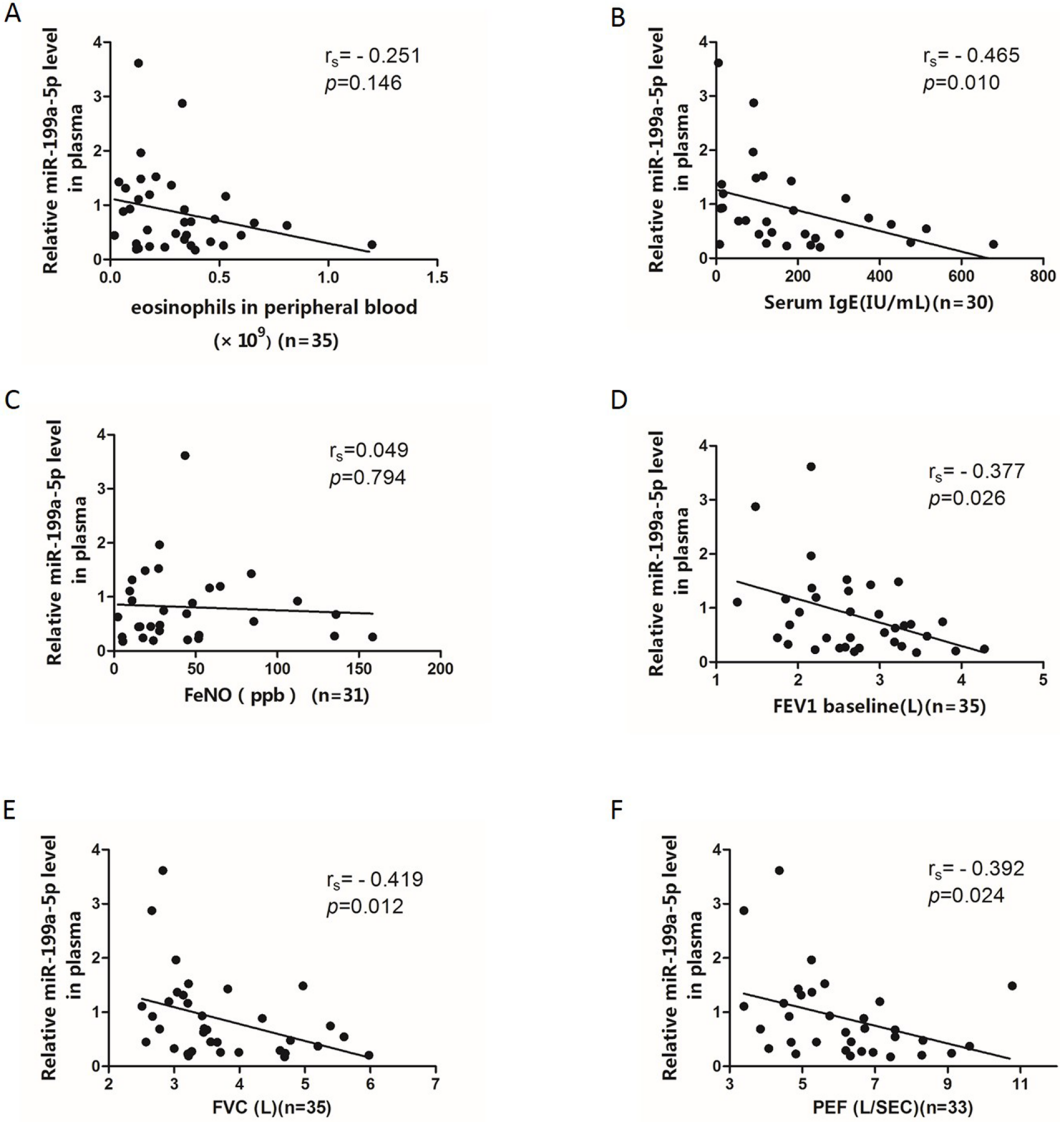
Correlations between the plasma miR-199a-5p levels and clinical indexes. (A-F) Spearman correlation between expression of miR-199a-5p in plasma and eosinophils in peripheral blood (A), serum IgE levels (B), FeNO levels (C), FEV1 (D), FVC (E), and PEF (F).

We further clarified the relationships between expression of plasma miR-199a-5p with the blood eosinophil count, serum IgE level, FeNO, FEV_1_, FVC and PEF between asthmatic cases using Spearman correlation analysis (Fig 5). The plasma miR-199a-5p levels were significantly and negatively correlated with serum IgE levels (r = -0.465, *p* = 0.010), which indicates its relationship to non-atopic asthma. Plasma miR-199a-5p was negatively correlated with FEV_1,_ FVC and PEF (r = -0.377, *p* = 0.026; r = -0.419, *p* = 0.012; r = -0.392, *p* = 0.024, respectively). Multivariate correlation analysis confirmed that plasma miR-199a-5p levels were negatively correlated with FEV_1_ in patients with asthma (Adjusted R^2^ = 0.164, *p*(model) = 0.015; β = -0.439, *p* = 0.015, Table 2).

**Table 2:** Multiple linear regression analysis of the factors related with miR-199a-5p expression.

miRNA ID	Adjusted *R*^2^	*P* value model	Predictor variable	β	*P* value
Multiple linear regression (stepwise method)					
miR-199a-5p	0.164	**0.015**	Age	0.211	0.304
			BMI	-0.32	0.064
			Atopy	0.167	0.968
			Sex	-0.074	0.749
			FEV_1_	-0.439	**0.015**
			Blood eosinophils count	-1.778	0.087
			Serum IgE	-1.924	0.065
			Pack years	0.024	0.891

Multiple regression analysis with age, BMI, atopy, sex, FEV1, blood eosinophils count, serum IgE and pack years as predictor variables, followed by stepwise multiple linear regression.

### In silico analysis into the functional role of miRNA

**Fig 6:**
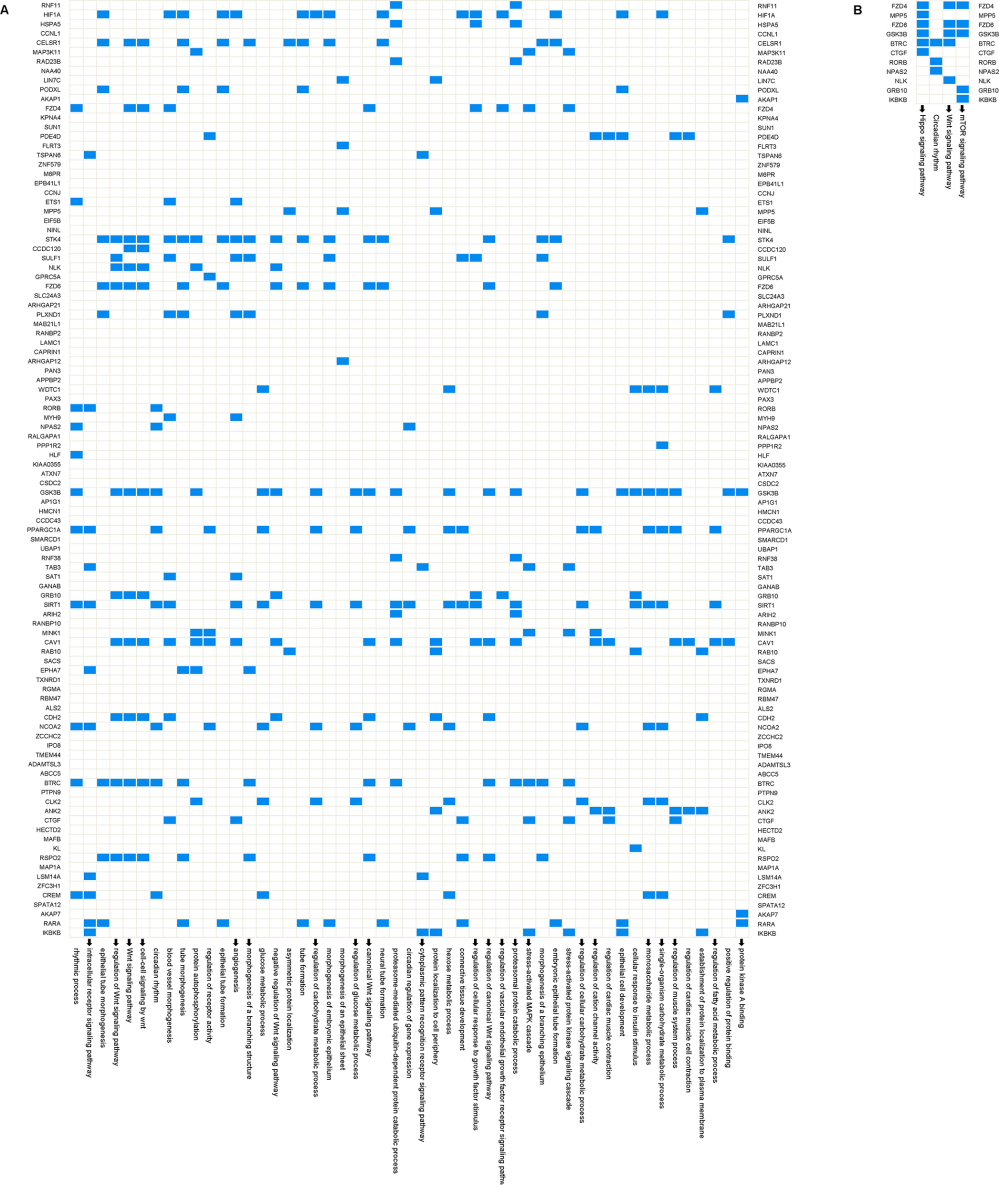
In silico analysis into the functional role of miRNA. Overview of the GO(A) and KEGG(B) pathways that contain at least 1 of the genes targeted by miR-199a-5p. Target genes are indicated on both sides. Targets expressed in the pathway are indicated in blue, and white indicates that the gene was not expressed in the gene set. Arrows represent pathways with potential interest in the proliferation of smooth muscle cells.

We used the TargetScan, miRanda and PicTar databases to predict the target genes of miR-199a-5p and selected the common genes. This procedure generated 100 potential target genes (Fig 6A). We selected genes within all of the Gene Ontology (GO) and Kyoto Encyclopedia of Genes and Genomes (KEGG) pathway gene sets [33, 34] that contained at least 1 of the 100 potential target genes, and 51 and 4 pathways were identified, respectively(Fig 6). In silico analysis predicted the validated and high score miR-199a-5p target genes and implicated the WNT, mTOR and hippo signaling pathways as the major miR-199a-5p-regulated pathways. The WNT signaling pathway was extremely important for miR-199a-5p mediation of hypertrophy and proliferation of smooth muscle cells.

### miR-199a-5p inhibited WNT expression in HASMCs

**Fig 7:**
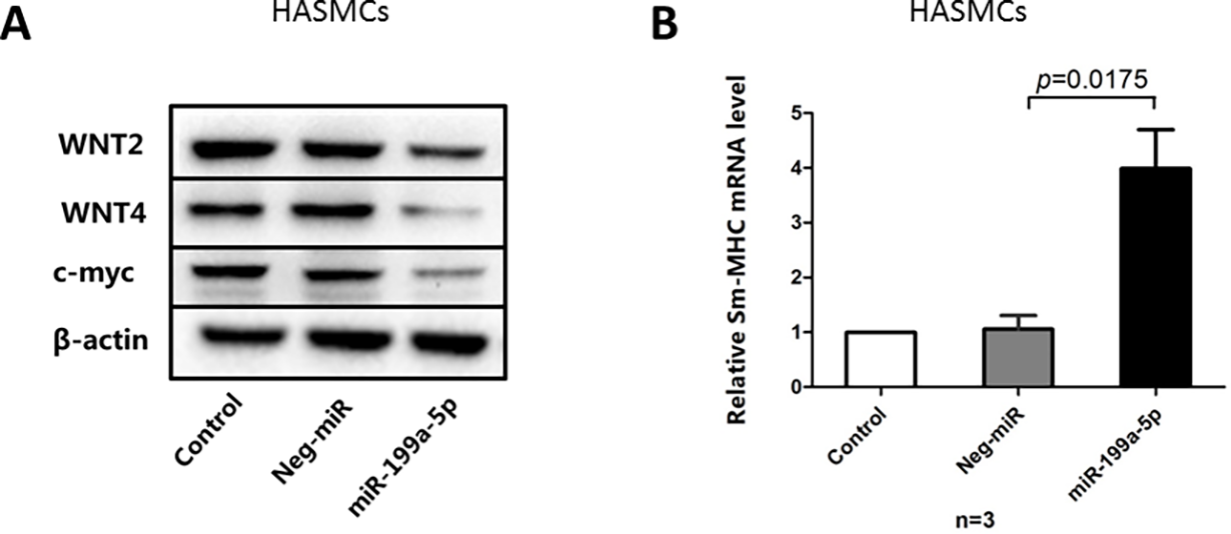
miR-199a-5p modulates the WNT signaling pathway and increases mRNA expression of Sm-MHC. (A) Western blot analysis of WNT2, WNT4, and c-myc in untreated (control) and negative miRNA (Neg-miR) and miR-199a-5p mimics transfection (miR-199a-5p) for 48hours in HASMCs. (B) Relative mRNA level of Sm-MHC in untreated control, negative miRNA and miR-199a-5p mimics transfection of HASMCs.

To investigate the role of miR-199a-5p in modulating human airway smooth muscle function, we transfected HASMCs with miR-199a-5p mimics and negative control. As shown in Fig 7A, after transfection of miR-199a-5p mimics for 48 hours, the protein expressions of both WNT2 and WNT4 were inhibited in HASMCs compared to that of the negative control. In the meantime, the protein expression of the cell growth protein c-myc decreased in the miR-199a-5p mimics group compared with the negative control. On the contrary, the expression of Sm-MHC mRNA of HASMCs significantly increased in the miR-199a-5p mimics group compared with the negative control (Fig 7B), indicating miR-199a-5p may modulate the hypertrophy and over contraction of HASMCs.

## Discussion

This study identified upregulation of miR-199a-5p expression in plasma and induced sputum of patients with the neutrophilic phenotype of asthma. None of the included patients were treated with inhaled or oral corticosteroids in the past three months to avoid the influence of steroids on the neutrophil counts and miR-199a-5p expression. Linear regression analysis demonstrated that expression of miRNA in plasma was negatively associated with the serum IgE levels. The plasma miR-199a-5p level was significantly associated with impaired pulmonary function (FEV_1_, FVC, and PEF). We demonstrated that miR-199A-5p was only induced in neutrophils, but not macrophages, lymphocytes, or epithelial cells. In silico analysis indicated that the identified miR-199a-5p may regulate pathways related to the inflammation response and muscle cell hypertrophy.

To our knowledge, this study is the first report to describe a link between circulation miR-199a-5p expression in plasma and patients with neutrophilic asthma. Patients with a neutrophil phenotype are more inclined to exhibit a poor response to corticosteroids and develop impaired pulmonary function with symptoms persisting [35]. Neutrophililic asthma patients require much more attention from health care workers, but there is almost a complete lack of a convenient and reliable biomarker to quickly identify neutrophilic asthma. Induced sputum and differential cell counts are mostly used to screen the neutrophil phenotype of asthma. Induced sputum, which enables the monitoring of genetic, biochemical, molecular, and cellular markers in proximal airways, is widely used in clinical practice and asthma research [36, 37]. However, not all asthma patients can successfully induce sputum, and a certain percentage of asthma patients are not suitable or cannot endure induced sputum [38]. The neutrophil phenotype of asthma has few of other options compared to the eosinophil phenotype of asthma, for which there are several alternate noninvasive biomarkers [39]. Circulation miRNAs are detected in certain disease contexts, including malignant tumors, cardiac disease, immune disorder sand asthma [7]. Expression of miRNAs in relation to the eosinophil count and atopic symptoms [24] suggests the use of circulation miRNAs as novel biomarkers to identify the phenotype of asthma. However, there are no related reports of a close relationship of circulation miRNAs to the neutrophil phenotype of asthma. We detected miR-199a-5p in plasma and found that it elevated in asthma patients with neutrophil infiltration in induced sputum, which indicates its potential to identify the neutrophil phenotype of asthma.

The most important characteristic of asthma is chronic airway inflammation. Therefore, we detected sputum miR-199a-5p and compared plasma expression and airway sputum expression of miR-199a-5p. The results demonstrated increased sputum miR-199a-5p in neutrophil asthmatics. Some reports indicated that circulation miRNAs did not necessarily parallel expression of local miRNAs [8], but the present study demonstrated that plasma miR-199a-5p was highly related with sputum miR-199a-5p. These results indicate that miR-199a-5p may participate in the communication between airway and systemic inflammation.

The expression profile of circulation miRNAs in relation to different pathophysiological conditions exhibited a specific pattern, which indicates that circulation miRNAs may not be passively released from necrotic or injured cells rather than selectively released from cells. The results of this study demonstrated that miR-199a-5p was increased following LPS stimulation in neutrophils, but not in macrophages, lymphocytes or epithelial cells, which supports that miR-199a-5p may be actively released by neutrophils and participate in the immunity disorder of the inflammation process of asthma.

The increased miR-199a-5p level was strongly related with impaired pulmonary function, which suggests that smooth muscle cell function was likely regulated by miR-199a-5p secreted from neutrophils. miR-199a-5p may mediate the neutrophil-smooth muscle cell communication and transform information from the inflammation response to a function change. Previous reports also demonstrated that the regulation of miR-199a-5p in muscle cells participated in cardiac remodeling and bladder organ remodeling [40, 41]. Increased miR-199a-5p expression in smooth muscle cells increases the synthesis of contractile and structural proteins and leads to smooth muscle cell hypertrophy.

Bioinformatics predictions of the validated and high scores of miR-199a-5p targets genes implicated the WNT, mTOR and hippo signaling pathways as the major miR-199a-5p-regulated pathways. The WNT signaling pathway plays a pivotal role in the mediation of miR-199a-5p in hypertrophy and proliferation of bladder smooth muscle cells [41]. The other possible candidate is SIRT1, which is a validated miRWalk 2.0 target of miR-199a-5p that was also downregulated in endothelial cells treated with a miR-199a-5p mimics [42]. SIRT1 was identified as a mediator of connective tissue development [43, 44]. In this study, we confirmed miR-199a-5p could inhibit WNT2/4 expression accompanied by decreased c-myc expression and increased Sm-MHC expression in human airway smooth muscle cells. Further research is needed to unravel the mechanism of miR-199a-5p regulation of the function and remodeling of airway smooth muscle in asthma.

One limitation of our study is that the cohort included a moderate number of patients with neutrophilic and eosinophilic asthma, which is a rather extinct phenotype of asthma. Paucigranulocytic and mixed granulocytic asthma were not included in this study because of our focus on neutrophilic asthma. Most published studies analyze more homogenous patient cohorts with mild, moderate to severe asthmatics. None of the patients in this study were treated or classified into various disease degrees of asthma.

The strong associations between circulation miR-199a-5p expression, inflammatory parameters and pulmonary function in patients with neutrophil phenotype asthma indicate that miR-199a-5p may contribute to the development of innate immunity and transfer the signal from inflammatory cells to structural cells in these asthma patients. Further research of the interactions between miR-199a-5p and airway smooth muscle cells may provide potential therapeutic targets for lung diseases characterized by neutrophilia and airway obstruction.

## Conclusions

Expression of circulation miR-199a-5p was increased in asthma patients with a neutrophil phenotype, which suggests that miR-199a-5p actively contributes to disease pathogenesis by modulating the proinflammatory process and pulmonary function.

## Supporting information

S1 Fig Wright-Giemsa or immunocytochemical staining on different cells isolated from peripheral blood. neutrophils (A) and lymphocytes (B) were isolated from peripheral blood, of which cytospins were prepared and stained with Wright-Giemsa. (C) Macrophages were obtained as described under “Materials and Methods” and were identified with immunohistochemical stain of CD68. Scale bar = 200 μm.
(TIF)

S1 File Supporting information: Database. (XLSX)
