# Supplementary figures and images for: Plasma miR-199a-5p is increased in neutrophilic phenotype asthma patients and negatively correlated with pulmonary function
Source: PLoS One. 2018 Mar 5;13(3):e0193502. doi: 10.1371/journal.pone.0193502 (PMC5837185; doi:10.1371/journal.pone.0193502)

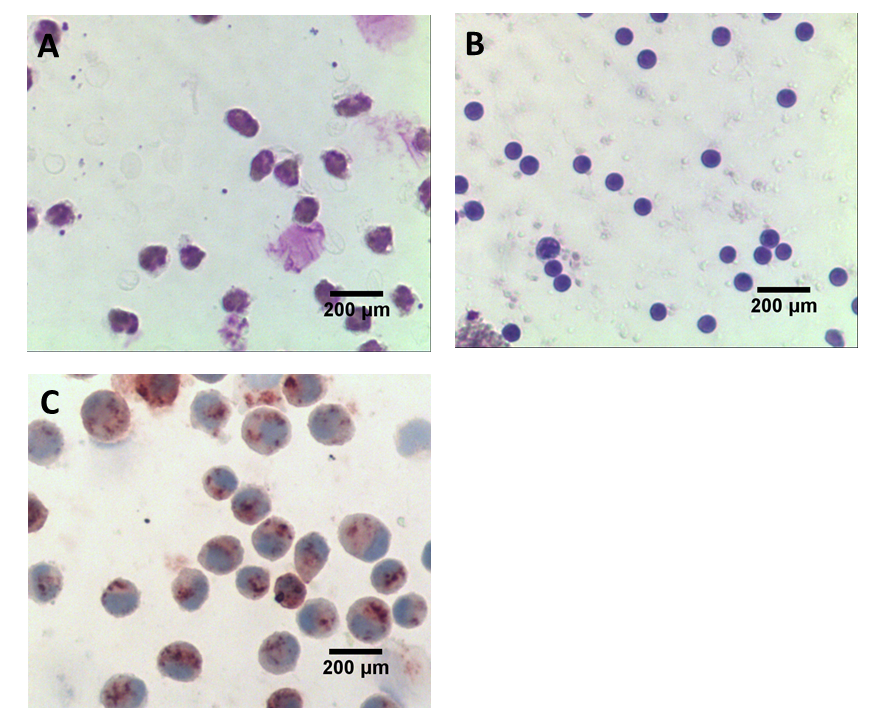

Supplement: S1 Fig — neutrophils (A) and lymphocytes (B) were isolated from peripheral blood, of which cytospins were prepared and stained with Wright-Giemsa. (C) Macrophages were obtained as described under “Materials and Methods” and were identified with immunohistochemical stain of CD68. Scale bar = 200 μm. (TIF) [file pone.0193502.s001.tif]
